# Modeling and Thermodynamic Studies of γ‐Valerolactone Production from Bio‐derived Methyl Levulinate

**DOI:** 10.1002/gch2.202200208

**Published:** 2023-02-22

**Authors:** Elena Montejano‐Nares, Francisco Ivars‐Barceló, Sameh M. Osman, Rafael Luque

**Affiliations:** ^1^ Departamento de Química Inorgánica y Química Técnica Facultad de Ciencias UNED Av. Esparta s/n Las Rozas de Madrid Madrid 28232 Spain; ^2^ Departamento de Química Orgánica Edif. Marie Curie Universidad de Córdoba Ctra Nnal IV‐A, Km 396 Córdoba E14014 Spain; ^3^ Chemistry Department College of Science King Saud University P.O. Box 2455 Riyadh 11451 Saudi Arabia; ^4^ Universidad ECOTEC Km 13.5 Samborondón Samborondón EC092302 Ecuador

**Keywords:** γ‐valerolactone, Methyl levulinate, Aspen Plus, Thermodynamic analysis, Hydrogenation

## Abstract

The exploitation of biomass to reduce the dependency on fossil fuels represents a challenge that needs to be solved as soon as possible. Nowadays, one of the most fashionable processes is γ‐valerolactone (GVL) production from bio‐derived methyl levulinate (ML). Deep understanding of the thermodynamic aspects involved in this process is key for a successful outcome, but detailed studies are missing in the existing literature. A thermodynamic study of the reaction of γ‐valerolactone (GVL) production from bio‐derived methyl levulinate (ML) is performed by the Gibbs free energy minimization method. The effect of various reaction conditions (temperature, concentration, flow rate) and the implication of possible intermediates and byproducts are assessed. Conversion and selectivity are calculated from the simulation of the ML hydrogenation using isopropanol as the hydrogen donor under continuous flow conditions. Significant increases in GVL selectivity can be achieved under dry conditions, keeping the high conversion. Comparison between theoretical and experimental results from a previous article discloses the effect of using 5%RuTiO_2_ catalysts, which increases the selectivity from 3–40% to 41–98%. Enthalpy and Gibbs free energy of the reactions at issue are also calculated from models using Barin equations according to Aspen Physical Property System parameters.

## Graphical abstract

Modeling and thermodynamic studies for γ‐valerolactone (GVL) production from bio‐derived methyl levulinate (ML), performed using Aspen Plus, point to an enhanced GVL selectivity under dry conditions with no significant influence on conversion. Thermodynamic parameters are also determined to support previously reported experimental results.

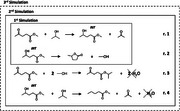

## 1 Introduction

At present, fossil‐based energy resources represent about three‐quarters of the world primary energy consumption.^[^ ^1^ ^]^ In the last years, the search for new energy sources is increasingly attracting the interest of the research community and industrial companies, driven by the need of substituting these traditional fuels associated with non‐sustainable development. Biomass is the only renewable energy resource capable of replacing fossil fuels, whose exploitation provides a favorable carbon dioxide balance.^[^ ^2^, ^3^, ^4^, ^5^, ^6^, ^7^ ^]^ Thus, the obtention of high‐valued biochemicals and liquid biofuels from biomass contributes to ensure environmental sustainability. In this sense, the implementation of innovative methodologies to accomplish the conversion of this versatile resource represents a challenge. Among all the biomass, lignocellulose, which consists of three main structural units (cellulose, hemicellulose, and lignin), is inexpensive, sustainable, and has widespread worldwide availability.^[^ ^8^, ^9^, ^10^, ^11^ ^]^ Several biological and chemical catalytic routes to transform lignocellulosic biomass have been reported, such as reactions of condensation,^[^ ^12^ ^]^ reduction,^[^ ^13^ ^]^ esterification,^[^ ^14^ ^]^ etherification,^[^ ^15^ ^]^ acetalization,^[^ ^16^ ^]^ hydrothermal carbonization,^[^ ^17^ ^]^ hydrolysis or hydrogenolysis,^[^ ^18^ ^]^ and what is called organosolv fractionation.^[^ ^19^ ^]^ Among the variety of substances that can be obtained from biomass, methyl levulinate (ML) as well as other levulinate esters can be used as gasoline and diesel additives, solvents, plasticizers, or flavorings thanks to their interesting properties.^[^ ^20^, ^21^ ^]^ Some of the strategies reported to obtain methyl levulinate from biomass are based on the development of acid (Brønsted and Lewis) catalytic systems which allow, for instance, direct liquefaction of bamboo,^[^ ^22^ ^]^ delignification of hydrothermal wood residue,^[^ ^23^ ^]^ or solvolysis of cellulose.^[^ ^24^ ^]^ In this study, the target is focused on the transformation of ML into the cyclic ester γ‐valerolactone (GVL), one of the highest value‐added chemicals.^[^ ^8^, ^25^, ^26^, ^27^ ^]^ GVL is an organic renewable component identified as a non‐toxic green solvent, fuel, and feedstock.^[^ ^28^, ^29^ ^]^ In fact, this substance is fully biodegradable and highly stable, so it is often used as a food and fragrance additive, among many other uses.^[^ ^30^, ^31^ ^]^ Its wide‐ranging applicability has led to the study of numerous hydrogenation catalysts for its production, either homogeneous or heterogeneous catalytic systems.^[^ ^32^, ^33^, ^34^, ^35^ ^]^ A recent work published by our group comprises the continuous‐flow catalytic transfer hydrogenation of industrially derived ML biowaste (from Avantium Chemicals B.V.) to form GVL by using a series of versatile Ru/TiO_2_ catalysts; and the study of the effects of several reaction conditions (e.g., temperature, concentration and flow rate) on ML conversion and GVL selectivity.^[^ ^36^ ^]^ Instead of H_2_ or formic acid, 2‐propanol was used as solvent, which is reported to be an active hydrogen source.^[^ ^37^, ^38^, ^39^ ^]^ With reference to the reactor setups, most of the reported works dealing with hydrogenation of levulinic acid to form GVL employ batch conditions.^[^ ^40^, ^41^, ^42^ ^]^ However, continuous flow reactor setups bring numerous advantages regarding space‐to‐time yield productivity, purification, environmental impact, and easy scale‐up, among others.^[^ ^43^, ^44^ ^]^ For that reason, although efficient flow processes for GVL obtention at low energy expenses have not been implemented yet,^[^ ^42^ ^]^ this study intends to approach that perspective, trying to mimic what could become a large‐scale production process. The aim of the present work is to perform a detailed modeling and thermodynamic study assessing and comparing it with the experimental results, to shed more light on the mechanisms of the reaction at issue.

## 2 Methodology and Model Description

The thermodynamic study of the reaction of methyl levulinate (ML) to produce γ‐valerolactone (GVL) was carried out by means of simulations conducted in Aspen Plus software. The Peng‐Robinson (PENG‐ROB) property base method was used to calculate the thermodynamic and transport properties of the components.^[^ ^45^, ^46^ ^]^ The thermodynamic properties are fugacity coefficient, enthalpy, entropy, Gibbs free energy, and volume; and the transport properties are viscosity, thermal conductivity, diffusion coefficient, and surface tension. This PENG‐ROB method is recommended for hydrocarbon and many gas processing applications. Still on this subject, Ariba et al.^[^ ^47^ ^]^ carried out physicochemical measurements (viscosity, density, refractive index, and specific heat capacity) of the substances involved in the hydrogenation of levulinic acid or its esters to produce GVL. In their thermodynamic model performed by Aspen Plus, they found that Hysys Soave‐Redlich‐Kwong (HYSSRK) and Benedict−Webb−Rubin−Starling (BWRS) models were suitable to describe their systems. Both methods, HYSSRK and BWRS, provide comparable results to the PENG‐ROB ones for phase equilibrium calculations. Nevertheless, the HYSSRK application range is significantly more limited, and BWRS results are more accurate for molar volume and enthalpy of liquids which is not our case.

The simulations were accomplished by using the RGibbs module which minimizes the Gibbs free energy (subject to atom balance constraints), in order to establish the equilibrium state at the specified temperatures and pressure for a given set of species without any specification of the possible reactions taking place in the system.^[^ ^49^ ^]^ This reactor can model single‐phase chemical equilibrium and simultaneous phase and chemical equilibria.

**Figure 1:**
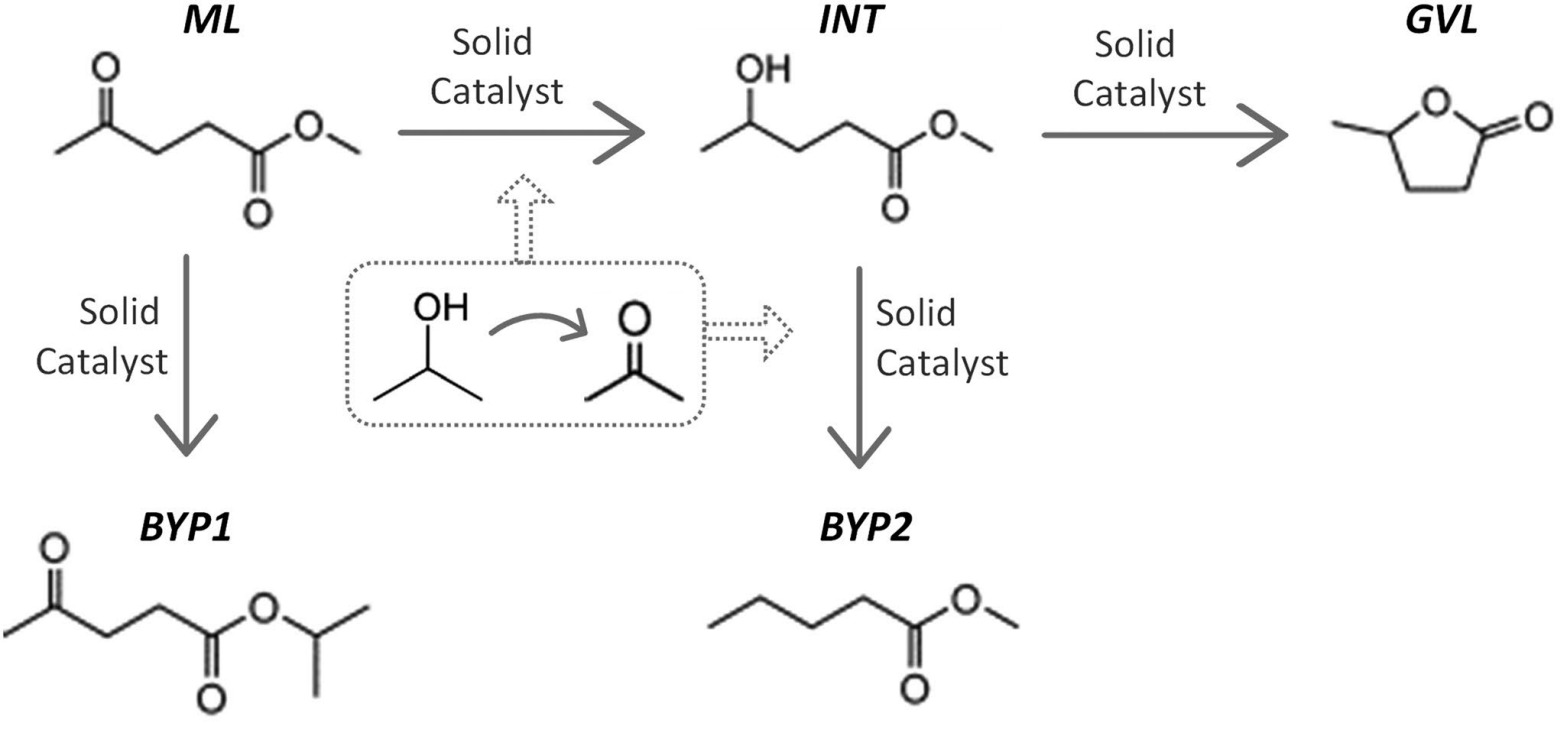
Possible reaction pathway for the continuous‐flow catalytic transfer hydrogenation of methyl levulinate by using Ru catalysts.^[^ ^36^ ^]^

**Figure 2:**
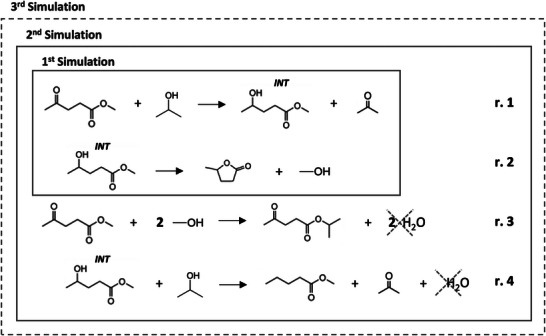
Simulation schemes and associated reactions considered for theoretical calculation using Aspen Plus. All reactions included in each simulation scheme have been treated as simultaneous possibilities: ML hydrogenation with isopropanol (**r. 1**), GVL production from the intermediate (**r. 2**), and byproducts production from ML (**r. 3**) and the intermediate (**r. 4**). Acronyms: INT = Intermediate (methyl 4‐hydroxypentanoate).

**Figure** **1** shows all the chemical species and possible reaction pathways of the process at issue reported in an article recently published by our group.^[^ ^36^ ^]^ All these reported species have been considered in the thermodynamic models of the present work and are collected in **Table** **1**. Following Figure 1, ML would react with isopropanol to form the intermediate methyl 4‐hydroxypentanoate (INT) which, in turn, would give rise to GVL and methanol, as widely established.^[^ ^27^, ^49^, ^50^, ^51^, ^52^ ^]^ Despite this main pathway, other byproducts were experimentally detected, 2‐propyl levulinate and methyl pentanoate, which were suggested to be formed from the ML and the hydrogenated intermediate, respectively. In this work, for the purpose of further analysis, such reactions were adjusted by including the species required to meet the stoichiometry (**Figure** **2**).

**Table 1:** Species considered in the simulation models

Name(s)	Alias	Molecular formula	CAS Registry Number	Structure
Methyl Levulinate	ML	C_6_H_10_O_3_	624‐45‐3	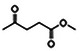
2‐Propanol	ISOP	C_3_H_8_O	67‐63‐0	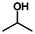
Methyl 4‐hydroxypentanoate (Valeric acid)	INT	C_6_H_12_O_3_	85433‐45‐0	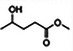
2‐Propyl levulinate (1‐Methylethyl 4‐oxopentanoate)	BYP1	C_8_H_14_O_3_	21884‐26‐4	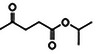
Methyl pentanoate (Methyl valerate)	BYP2	C_6_H_12_O_2_	624‐24‐8	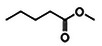
γ‐Valerolactone	GVL	C_5_H_8_O_2_	108‐29‐2	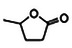
Acetone	ACE	C_3_H_6_O	67‐64‐0	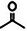
Methanol	MetOH	CH_4_O	67‐56‐1	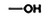
Water	H2O	H_2_O	7732‐18‐5	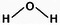

Aspen Plus components databases contain all the species introduced in the system (collected in Table 1), except for the intermediate methyl 4‐hydroxypentanoate, which was created from its molecular structure and whose properties were estimated by the software.

No catalyst was considered for the thermodynamic modeling since the catalyst employed for obtaining the experimental results, which are going to be used for comparison, was a solid.

The results obtained in all the simulations were compared by assessing the molar flow rate of the species under study at the outlet of the RGibbs module, calculating the conversion of ML (Equation 1) and the selectivity to GVL (Equation 2) as follows:

(1) $$\begin{array}{c} \text{Conversion}\left(\%\right)=\frac{FR_{\text{ML,initial}}-FR_{\text{ML,final}}}{FR_{\text{ML,initial}}}\times 100 \end{array}$$

(2) $$\begin{array}{c} \text{Selectivity}\left(\%\right)=\frac{FR_{\text{GVL,final}}\cdot \left(\frac{n_{\text{GVL}}}{n_{\text{ML}}}\right)}{FR_{\text{ML,initial}}-FR_{\text{ML,final}}}\times 100 \end{array}$$

where *FR*_ML,initial_ and *FR*_ML,final_ are the initial and final molar flow rates (mol·min^−1^) of the reagent ML, respectively; *FR*_GVL,final_ is the final molar flow rate (mol·min^−1^) of the product GVL; and *n*_GVL_ and *n*_ML_ are the number of carbon atoms in GVL (*n*_GVL_ = 5) and ML (*n*_ML_ = 6), respectively. It should be noted that, although the reagents were introduced in the reactor in a liquid‐phase continuous flow, only a gas‐phase was obtained at the outlet of the reactor for all the cases.

As mentioned before, Figure 2 displays the reactions that could take place in the system according to the reaction pathway shown in Figure 1. In line with these four reactions proposed, different simulations were carried out.

According to Figure 2, the 1^st^ simulation only considers the possibility of reactions **r. 1** and **r. 2** taking place, which implies that the byproducts, BYP1 and BYP2, are not obtained from ML and INT, respectively, and water does not form either. The 2^nd^ and 3^rd^ simulations conceive the four reactions as possible paths, but the latter does not consider the presence of water in the system. Once again, it should be pointed out that RGibbs module does not require any specification of the possible reactions taking place (neither the stoichiometry nor what is the reagent, product, or intermediate). In other words, there is freedom in the formation/consumption of any species as long as they have been included in the system, and the reaction at issue is thermodynamically feasible.

The three simulations in Aspen Plus have been conducted within the 100–200 °C temperature range using different inlet flow rates, between 0.3 and 1 mL·min^−1^, as well as different initial ML concentrations, between 0.3 and 0.6 mol·L^−1^, setting a pressure of 35 bar.

## 3 Results and Discussion

**Figure 3:**
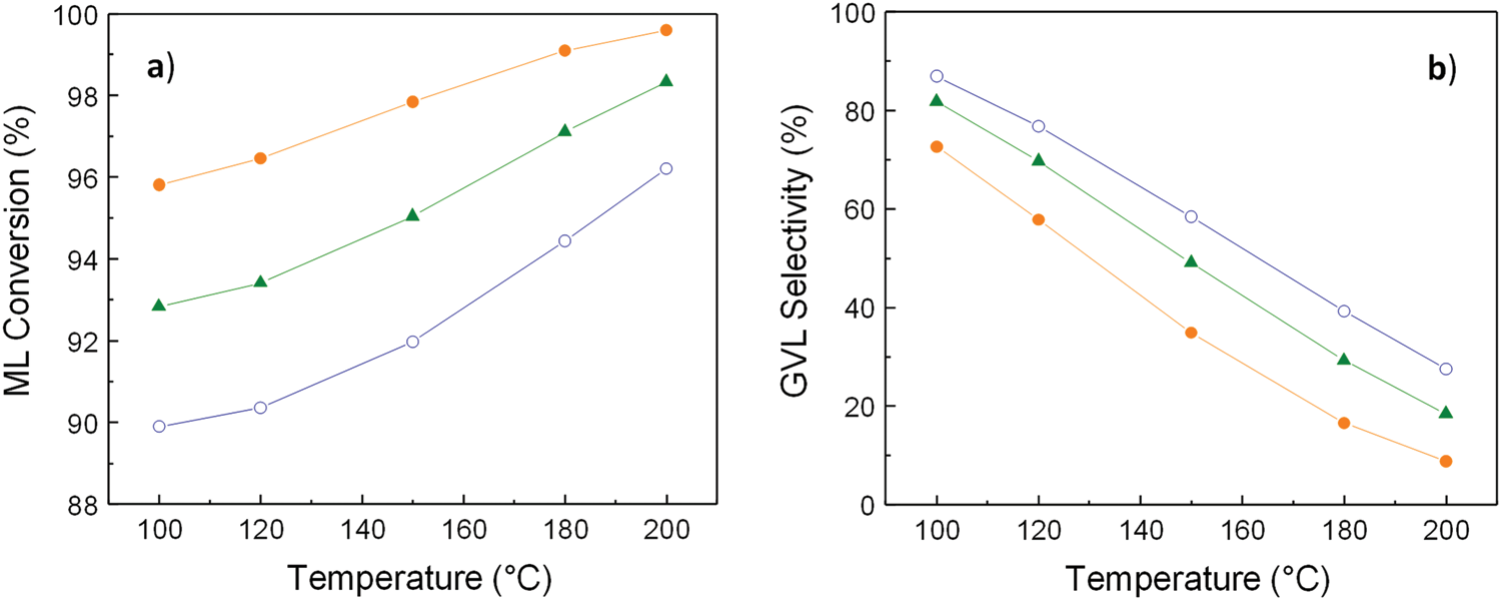
Conversion of ML a) and selectivity to GVL b) at a pressure of 35 bar for the 1^st^ simulation (Figure 2) conducted in Aspen Plus. Symbols: (‐●‐) C_ML,initial_ = 0.3 mol·L^−1^, (‐▲‐) C_ML,initial_ = 0.45 mol·L^−1^ (‐○‐), C_ML,initial_ = 0.6 mol·L^−1^ (for all initial flow rates between 0.3‐ 1 mL·min^−1^). Acronyms: C_ML, initial_ = ML initial concentration.

**Figure 4:**
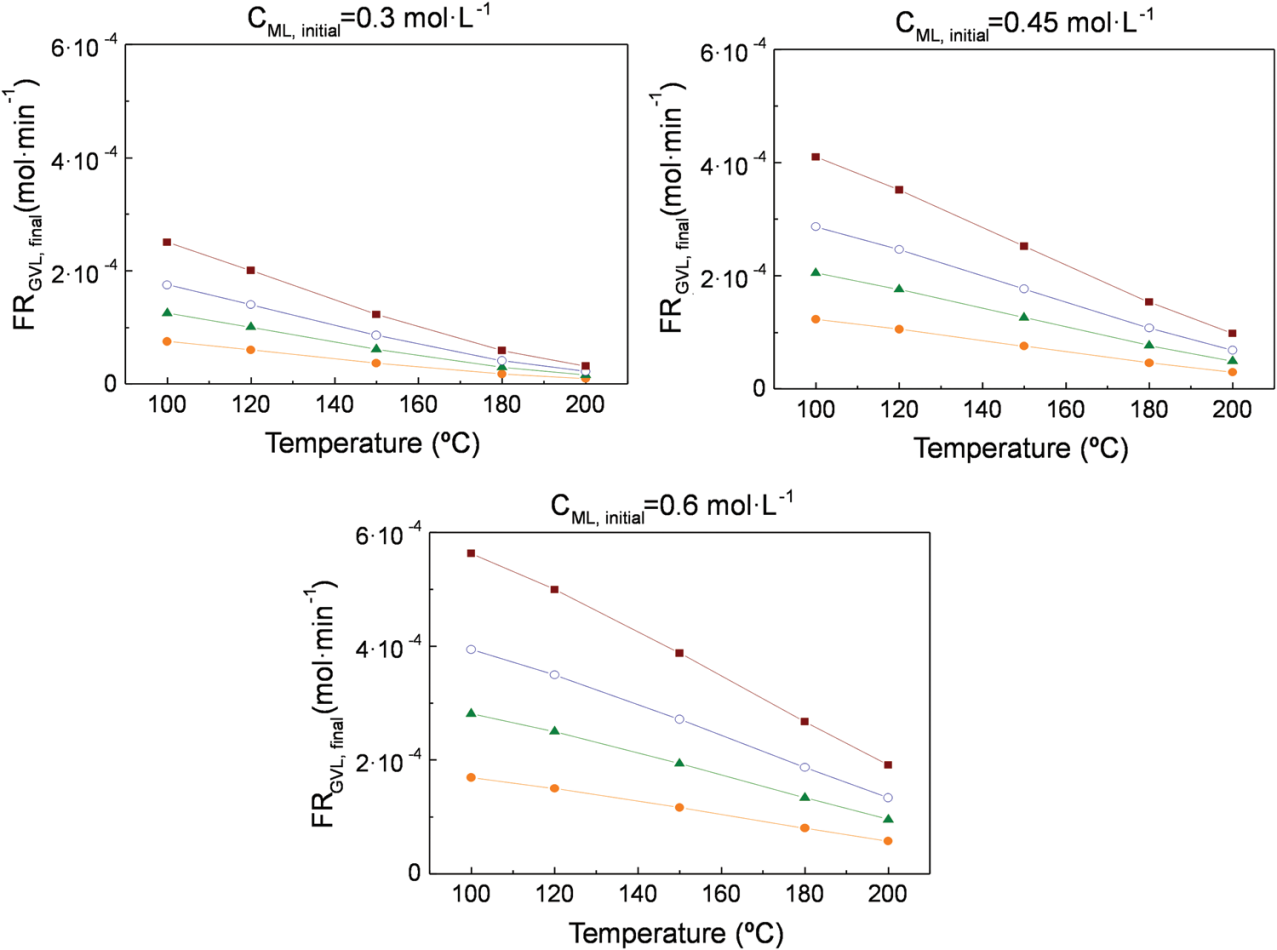
Molar flow rate of GVL at a pressure of 35 bar for the 1^st^ simulation (Figure 2) conducted in Aspen Plus. Symbols: (‐●‐) FR = 0.3 mL·min^−1^, (‐▲‐) FR = 0.5 mL·min^−1^ (‐○‐), FR = 0.7 mL·min^−1^, (‐∎‐) FR = 1 mL·min^−1^. Acronyms: FR_GVL,final_ = GVL final production, FR = Inlet flow rate, C_ML,initial_ = ML initial concentration.

**Figure 5:**
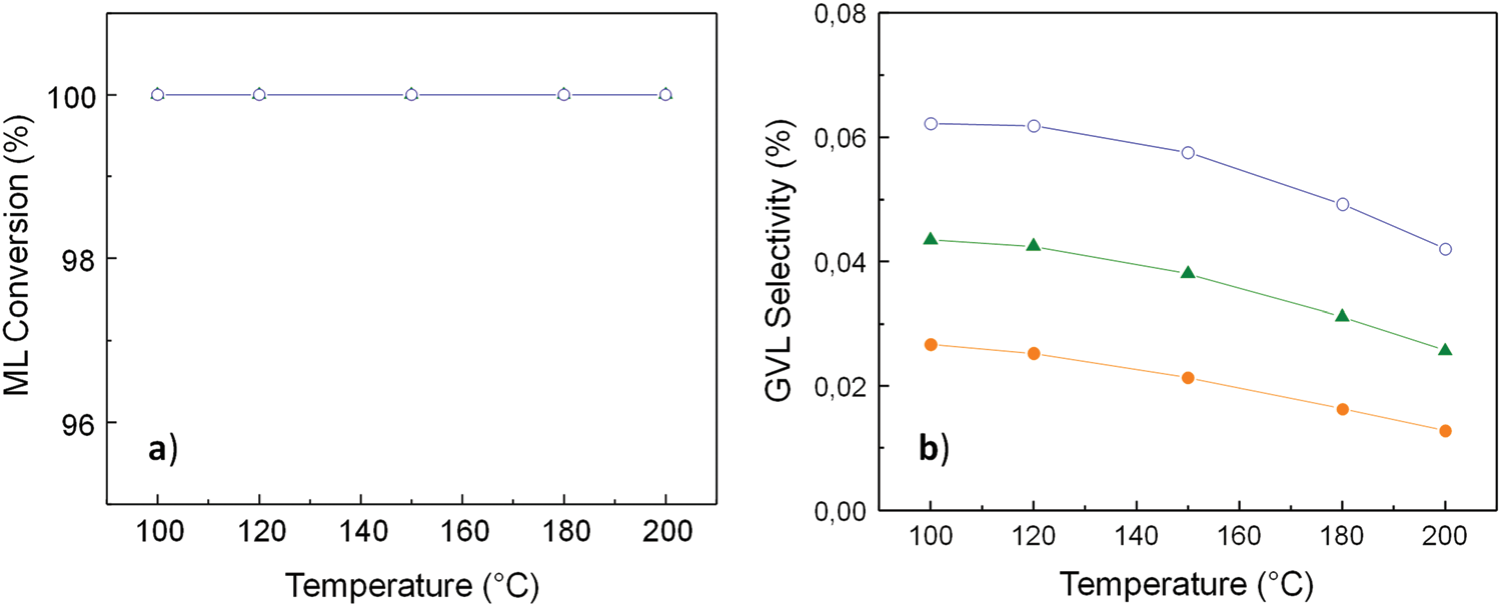
Conversion of ML a) and selectivity to GVL b) at a pressure of 35 bar for the 2^nd^ simulation (Figure 2) conducted in Aspen Plus. Symbols: (‐●‐) C_ML,initial_ = 0.3 mol·L^−1^, (‐▲‐) C_ML,initial_ = 0.45 mol·L^−1^ (‐○‐) C_ML,initial_ = 0.6 mol·L^−1^ (for all initial flow rates between 0.3‐ 1 mL·min^−1^). Acronyms: C_ML,initial_ = ML initial concentration.

**Figure 6:**
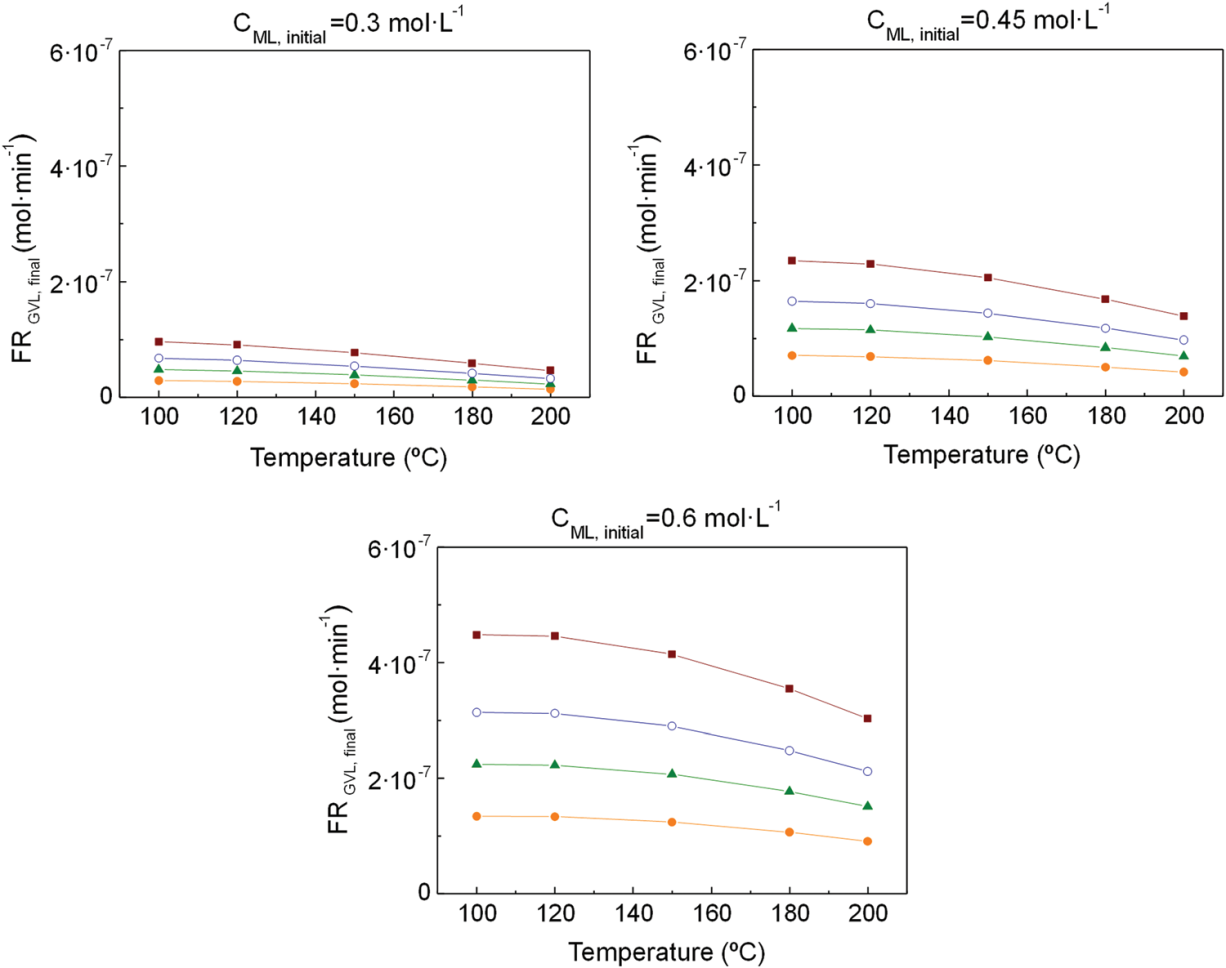
Molar flow rate of GVL at a pressure of 35 bar for the 2nd simulation (Figure 2) conducted in Aspen Plus. Symbols: (‐●‐) FR = 0.3 mL·min^−1^, (‐▲‐) FR = 0.5 mL·min^−1^ (‐○‐), FR = 0.7 mL·min^−1^, (‐∎‐) FR = 1 mL·min^−1^. Acronyms: FR_GVL, final_ = GVL final production, FR = Inlet flow rate, C_ML, initial_ = ML initial concentration.

**Figure 7:**
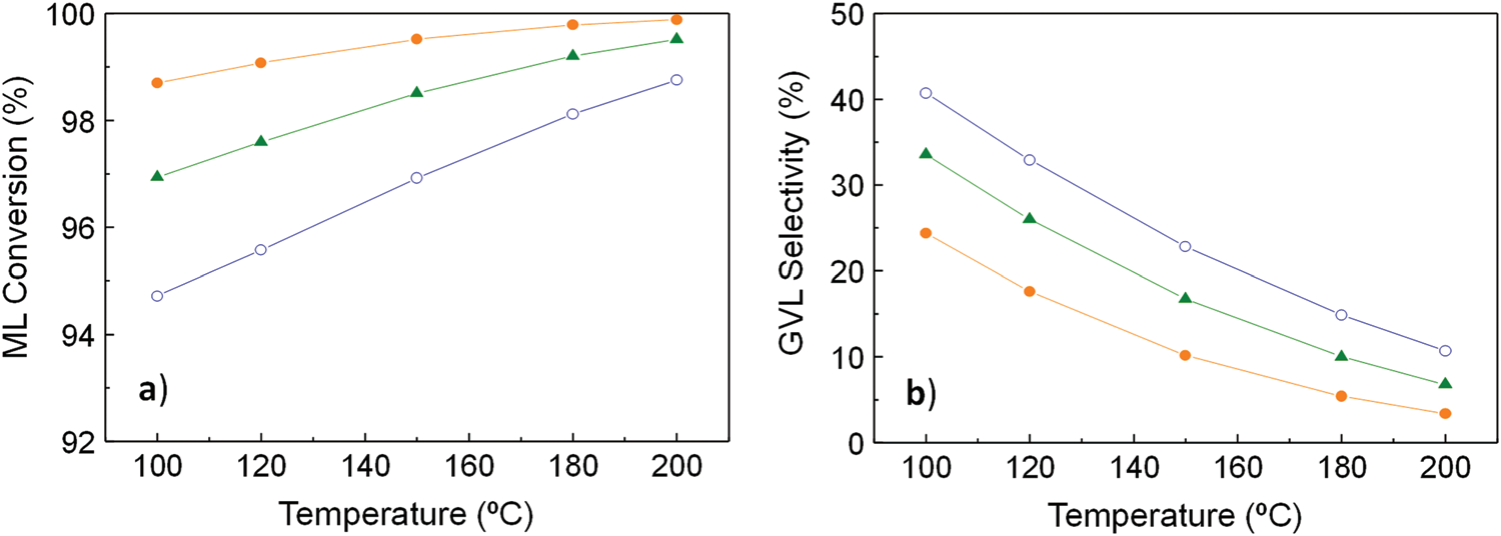
Conversion of ML **a)** and selectivity to GVL **b)** at a pressure of 35 bar for the 3^rd^ simulation (Figure 2) conducted in Aspen Plus. Symbols: (‐●‐) C_ML,initial_ = 0.3 mol·L^−1^, (‐▲‐) C_ML,initial_ = 0.45 mol·L^−1^ (‐○‐) C_ML,initial_ = 0.6 mol·L^−1^ (for all initial flow rates between 0.3‐ 1 mL·min^−1^). Acronyms: C_ML, initial_ = ML initial concentration.

**Figure 8:**
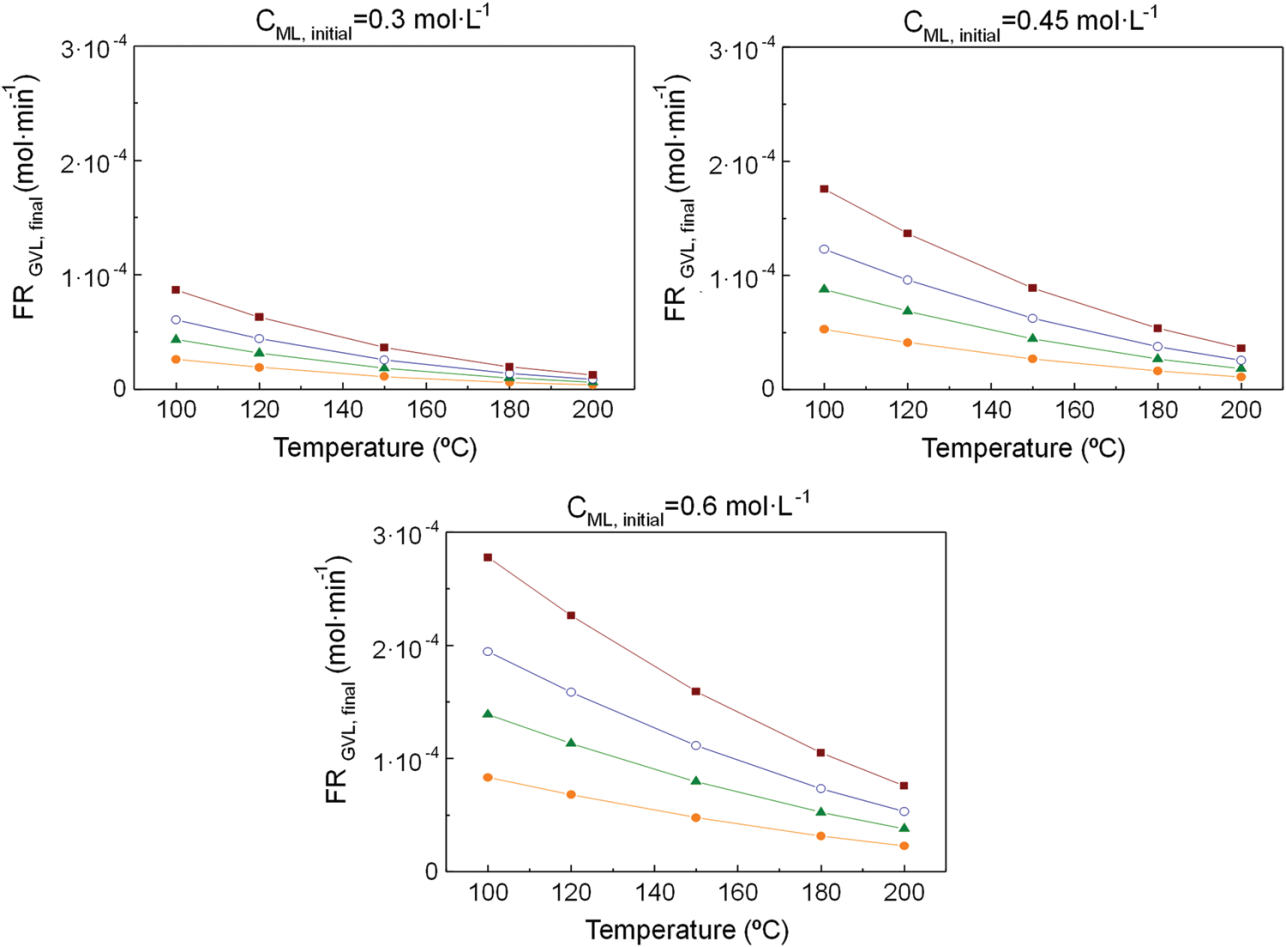
Molar flow rate of GVL at a pressure of 35 bar for the 3rd simulation (Figure 2) conducted in Aspen Plus. Symbols: (‐●‐) FR = 0.3 mL·min^−1^, (‐▲‐) FR = 0.5 mL·min^−1^ (‐○‐), FR = 0.7 mL·min^−1^, (‐∎‐) FR = 1 mL·min^−1^. Acronyms: FR_GVL,final_ = GVL final production, FR = Inlet flow rate, C_ML,initial_ = ML initial concentration.

For all the simulations, conversion and selectivity do not depend on the inlet flow rate (**Figures** **3**, **5**, **7**), but GVL production increases with it (**Figures** **4**, **6**, **8**). There are differences in the influence of the inlet flow rate on the GVL production depending on the simulation strategy (Figure 2), although the rise in the production does not overcome one order of magnitude in any case.

Regarding the 1^st^ simulation (considering only the reactions of GVL production from ML), it is observed that high temperatures and low ML initial concentrations give rise to higher conversions (Figure 3a), as would be expected. These conversions start in values ≈90%, for the lowest temperature and highest ML concentration, and reach ≈100%, for the highest temperature (200 °C) and lowest ML concentration (0.3 mol·L^−1^). By contrast, the increase in temperature and decrease in ML concentration prompt a decline in GVL production (Figure 4) and, thus, in GVL selectivity values, moving from 90% to 10% (Figure 3b). In relation to the starting basis of this simulation, it should be noted that since the species involved in the secondary reactions are not included in the system, the reagents are limited in terms of transformation into products. For this reason, this case can be considered as an ideal scenario thanks to which implications of the presence or absence of byproducts can be evaluated.

The results of the 2^nd^ simulation (considering as possible products all the species involved in the four reactions) show a total conversion regardless of temperature, initial inlet flow rate, and ML concentration (Figure 5a). As well as in the previous scenario, the tendency of GVL formation and selectivity declines with temperature and low ML initial concentrations (Figures 5, 6), but in this simulation, these values have drastically dropped. This remarkable decrease in GVL formation shifts from 10^−4^ (1^st^ simulation) to 10^−7^ (2^nd^ simulation) mol·min^−1^ which results in <0.1% of selectivity, and it is associated with a high production of water and the byproduct methyl pentanoate (Supporting Information: Figure S2), whose orders of magnitude are ≈10^−4^ mol·min^−1^ (Supporting Information: Table S2).

Finally, the 3^rd^ simulation (considering all the species involved in the four reactions except water) could be conceived as the most realistic scenario because the experimental reaction is performed by using a desiccant. The results present the same graphic trends as seen previously in the other scenarios: conversion rises with temperature and drops with ML concentration (Figure 7a), and the reverse occurs for GVL formation (Figure 8) and selectivity (Figure 7b). The conversion values vary from 95 to 100% and the selectivity ones, from 3 to 40%. This selectivity wide range reveals that ML concentration and, with greater influence, temperature are key factors that have a big effect on the reaction, and, thus, must be largely taken into account. The order of magnitude for GVL formation is around 10^−4^ mol·min^−1^, which, in combination with the other outcome, makes the results of this last scenario more similar to the ones obtained in the 1^st^ simulation.

**Table** **2** collects the results of conversion and selectivity achieved experimentally by using 5%Ru/TiO_2_ as catalyst,^[^ ^36^ ^]^ and the results obtained theoretically through the three simulations conducted in Aspen Plus. Although both outcomes cannot be directly compared since the experimental reactions were performed using catalysts, some considerations might be discussed in terms of the involvement of the species, the influence of some reaction parameters, and the catalyst effect. The greater similarity between the experimental conversion and that derived from the simulation not taking into account byproducts and water formation (1^st^ Simulation in Figure 2), may suggest that the equilibrium could be more shifted to the right in reactions **r. 1** and **r. 2** (Figure 2), making them more favored. On the other hand, the discrepancies between the experimental and theoretical selectivity values, allow identifying the effect of the catalyst presence. In this sense, comparing the theoretical selectivity calculated from the 3^rd^ simulation (in absence of catalysts) with the available experimental selectivity values (derived from the use of 5%Ru/TiO_2_ catalysts), it can be observed that the range increases from 3–40% (Figure 7b) to 41–98% (Table 2), respectively. This rise reveals the exceptional catalytic performance that this 5%Ru/TiO_2_ material presents in the target reaction. In addition, theoretical selectivity (Figures 3, 5, 7) decreases with conversion as the temperature increases, in contrast with the increasing selectivity values experimentally obtained employing a catalyst for 150, 180, and 200 °C in Table 2. Moreover, while theoretical selectivity remains constant to the increasing inlet FR (from 0.3 to 0.7 mL·min^−1^), the experimental selectivity drops with it (Table 2). The differences in selectivity between the theoretical and experimental results are due to the absence or presence of the catalyst, respectively.

**Table 2:** Comparison between Aspen Plus simulations and catalytic experiments for GVL transformation

			1^st^ Simulation			2^nd^ Simulation			3^rd^ Simulation			Experimental^a)^	
C_ML,initial_ [mol·L^−1^]	FR [mL·min^−1^]	T [°C]	Conv. [%)	Select. [%]		Conv [%]	Select. [%]		Conv. [%]	Select. [%]		Conv. [%]	Select. [%]
0.3	0.3	150	97.8	34.9		100	0.02		99.5	10.1		98	71
		180	99.1	16.6		100	0.02		99.8	5.4		98	98
		200	99.6	8.8		100	0.01		99.9	3.4		99	98
	0.5	200	99.6	8.8		100	0.01		99.9	3.4		90	41
	0.7	200	99.6	8.8		100	0.01		99.9	3.4		95	48
0.45	0.3	200	98.3	18.4		100	0.03		99.5	6.8		98	62
0.6	0.3	200	96.2	27.6		100	0.04		98.8	10.7		96	98

^a]^ Experimental results obtained for the reaction employing the 5%Ru/TiO_2_ catalyst.^[^ ^36^ ^]^ Acronyms: C_ML,initial_ = ML initial concentration, FR = initial flow rate, T = temperature.

Lastly, Aspen Plus was also employed for the obtention of thermodynamic data. The enthalpy of formation and Gibbs free energy of formation (**Table** **3**) for the substances involved in the reactions were obtained to calculate the reaction energies (**Table** **4**) in accordance with the stoichiometry of the reactions proposed in Figure 2.

**Table 3:** Enthalpy (∆*H*°_F_) and Gibbs free energy (∆*G*°_F_) for the reaction species formation at different conditions of temperature and pressure

	25 °C, 1 atm			100 °C, 35 bar			200 °C, 35 bar	
	∆*H*°_F_	∆*G*°_F_		∆*H*°_F_	∆*G*°_F_		∆*H*°_F_	∆*G*°_F_
Component alias^a)^	[kJ·mol^−1^]			[kJ·mol^−1^]			[kJ·mol^−1^]	
ML	−584.4	−440.0		−576.3	−402.4		−558.1	−357.2
ISOP	−272.4	−175.3		−271.9	−145.0		−263.0	−110.7
INT	−643.6	−450.4		−634.8	−400.5		−615.1	−339.2
ACE	−216.0	−151.4		−216.1	−128.8		−208.2	−105.1
MetOH	−201.1	−162.4		−204.2	−144.7		−195.8	v128.7
BYP1	−654.6	−425.7		−642.7	−367.5		−618.3	v295.8
BYP2	−471.5	−310.9		−464.2	−267.6		−446.1	−216.2
GVL	−418.7	−297.3		−413.3	−264.1		−398.2	−225.2
H2O	−241.9	−228.6		−244.3	−215.9		−237.9	−209.0

^a)^ The names of the components associated with the alias presented in this table are collected in Table 1.

**Table 4:** Enthalpy of reaction (∆*H*°_R_) and Gibbs free energy of reaction (∆*G*°_R_) for the reactions proposed in Figure 2 under different conditions of temperature and pressure

	25 °C, 1 atm			100 °C, 35 bar			200 °C, 35 bar	
	∆*H*°_R_	∆*G*°_R_		∆*H*°_R_	∆*G*°_R_		∆*H*°_R_	∆*G*°_R_
Reactions	[kJ·mol^−1^]			[kJ·mol^−1^]			[kJ·mol^−1^]	
r. 1	−2.7	13.6		−2.6	18.0		−2.2	23.5
r. 2	23.7	−9.3		17.4	−8.3		21.2	−14.7
r. 3	−151.8	−118.1		−146.7	−107.5		−144.5	−99.2
r. 4	−13.4	−65.3		−17.8	−66.8		−14.0	−80.4

For most of the species and reactions under study, there are not much thermodynamic experimental data available. Geboers et al.^[^ ^53^ ^]^ obtained the energy values for reactions **r. 1** and **r. 2** from theoretical calculations by the G2(MP2) method, using the software Gaussian 09. The values of enthalpy at ambient conditions that they report are quite similar to the equivalent ones theoretically obtained in the present work (Table 4). Moreover, in this present work, the energy values have been also calculated for additional reaction conditions (35 bar, 100, or 200 °C) matching the ones employed in the experimental work.^[^ ^36^ ^]^ Enthalpies and Gibbs free energies of the reactions involving byproducts (**r. 3**, **r. 4**), never reported so far, have been also calculated here (Table 4). The results of the **r. 1** and **r. 2** enthalpies confirm the exothermicity and endothermicity of these reactions, respectively. Wang et al.^[^ ^54^ ^]^ also proved, through experimental measurements, the exothermicity and endothermicity of the hydrogenation and cyclization steps, respectively, although the enthalpy and Gibbs free energy values are not very similar. These discrepancies can lie in the fact that they employ different solvents and reaction conditions.

## 4 Conclusions

The thermodynamic study of the reaction of bio‐derived methyl levulinate (ML) to produce γ‐valerolactone (GVL) has been carried out by means of theoretical modeling conducted using the reference Aspen Plus software. From the reaction scenarios simulated and analyzed in this work, it has been determined that hydrogenation of ML, using isopropanol as the hydrogen source and by means of continuous flow conditions, gives rise to very high conversions (96–100%) regardless of byproducts formation including water. GVL selectivity tends to zero when water is produced and, hence, is present in the reaction system, while it rises up to 3–40% when water is removed. By comparing the theoretical modeling results (from the reaction in absence of a catalyst) with the experimental data (derived from the use of 5%Ru/TiO_2_ catalysts), it is observed that the selectivity range moves from 3–40% to 41–98%, respectively. These differences in the selectivity values allow to identify the catalyst effect, which in our case would be attributed to the catalyst used for the experimental results under comparison (5%Ru/TiO_2_). Theoretical GVL selectivity decreases with conversion as the temperature increases, in contrast with the experimental results employing the highly selective 5%Ru/TiO_2_ catalyst. The enthalpy and Gibbs free energy of the reactions have been calculated from Aspen Plus pure component energy data, revealing the exothermicity of the reaction **i**) ML to produce the intermediate (methyl 4‐hydroxypentanoate), and the endothermicity of the reaction **ii**) Intermediate to produce GVL.

## Conflict of Interest

The authors declare no conflict of interest.

## Supporting information

Supporting information

## Data Availability

The data that support the findings of this study are available from the corresponding author upon reasonable request.
