# Supplementary material for: Modeling and Thermodynamic Studies of γ‐Valerolactone Production from Bio‐derived Methyl Levulinate
Source: Glob Chall. 2023 Feb 22;7(4):2200208. doi: 10.1002/gch2.202200208 (PMC10069308; doi:10.1002/gch2.202200208)
Supplement: Supplementary file 1 — Supporting information [file GCH2-7-2200208-s001.pdf]

Global Challenges
Open Access

Supporting Information

for *Global Challenges,* DOI: 10.1002/gch2.202200208

Modeling and Thermodynamic Studies of γ-Valerolactone Production from Bio-derived Methyl Levulinate

Elena Montejano-Nares, Francisco Ivars-Barceló, Sameh M. Osman, and Rafael Luque**

SUPPORTING INFORMATION

Table S1
Concentration and flow rates of the species involved in the Aspen Plus 1st Simulation (**Fig. 2**) at the inlet and outlet of the reactor.

$C_{ML, initial}$ (mol/l)	$C_{ML, initial}$ (mol/min)	$C_{ISOP, initial}$ (mol/l)	$C_{ISOP, initial}$ (mol/min)	Initial Flow Rate (l/min)	T (°C)	$C_{ML, final}$ (mol/min)	$C_{ISOP, final}$ (mol/min)	$C_{INT, final}$ (mol/min)	$C_{GVL, final}$ (mol/min)	$C_{ACE, final}$ (mol/min)	$C_{MOH, final}$ (mol/min)
0.3	0.00009	10.987	0.00330	0.0003	100	3.77E-06	0.00319	3.21E-07	7.51E-05	1.19E-04	9.13E-05
					120	3.19E-06	0.00317	1.84E-07	6.02E-05	1.66E-04	9.98E-05
					150	1.94E-06	0.00313	7.17E-08	3.69E-05	2.41E-04	1.14E-04
					180	8.15E-07	0.00310	2.25E-08	1.78E-05	3.03E-04	1.25E-04
					200	3.67E-07	0.00309	9.03E-09	9.51E-06	3.30E-04	1.30E-04
	0.00015		0.00549	0.0005	100	6.28E-06	0.00532	5.36E-07	1.25E-04	1.98E-04	1.52E-04
					120	5.31E-06	0.00528	3.07E-07	1.00E-04	2.77E-04	1.66E-04
					150	3.24E-06	0.00522	1.20E-07	6.14E-05	4.02E-04	1.89E-04
					180	1.36E-06	0.00517	3.75E-08	2.96E-05	5.06E-04	2.08E-04
					200	6.12E-07	0.00514	1.51E-08	1.58E-05	5.50E-04	2.16E-04
	0.00021		0.00769	0.0007	100	8.79E-06	0.00745	7.50E-07	1.75E-04	2.77E-04	2.13E-04
					120	7.44E-06	0.00740	4.29E-07	1.41E-04	3.87E-04	2.33E-04
					150	4.54E-06	0.00731	1.67E-07	8.60E-05	5.63E-04	2.65E-04
					180	1.90E-06	0.00723	5.26E-08	4.14E-05	7.08E-04	2.91E-04
					200	8.57E-07	0.00720	2.11E-08	2.22E-05	7.70E-04	3.03E-04
	0.00030		0.01099	0.001	100	1.26E-05	0.01065	1.07E-06	2.50E-04	3.95E-04	3.04E-04
					120	1.06E-05	0.01057	6.13E-07	2.01E-04	5.53E-04	3.33E-04
					150	6.48E-06	0.01044	2.39E-07	1.23E-04	8.05E-04	3.78E-04
					180	2.72E-06	0.01033	7.51E-08	5.92E-05	1.01E-03	4.16E-04
					200	1.22E-06	0.01029	3.01E-08	3.17E-05	1.10E-03	4.32E-04

0.45	0.00013	10.728	0.00322	0.0003	100	9.68E-06	0.00309	7.28E-07	1.23E-04	1.30E-04	1.25E-04
					120	8.89E-06	0.00306	4.41E-07	1.05E-04	1.87E-04	1.36E-04
					150	6.69E-06	0.00301	2.01E-07	7.56E-05	2.86E-04	1.54E-04
					180	3.90E-06	0.00296	8.09E-08	4.60E-05	3.86E-04	1.74E-04
					200	2.26E-06	0.00293	3.93E-08	2.93E-05	4.43E-04	1.84E-04
	0.00022		0.00536	0.0005	100	1.61E-05	0.00515	1.21E-06	2.05E-04	2.17E-04	2.09E-04
					120	1.48E-05	0.00510	7.35E-07	1.76E-04	3.11E-04	2.26E-04
					150	1.12E-05	0.00502	3.34E-07	1.26E-04	4.76E-04	2.57E-04
					180	6.50E-06	0.00493	1.35E-07	7.67E-05	6.44E-04	2.89E-04
					200	3.77E-06	0.00488	6.55E-08	4.89E-05	7.38E-04	3.07E-04
	0.00031		0.00751	0.0007	100	2.26E-05	0.00721	1.70E-06	2.87E-04	3.04E-04	2.93E-04
					120	2.07E-05	0.00714	1.03E-06	2.46E-04	4.35E-04	3.17E-04
					150	1.56E-05	0.00703	4.68E-07	1.76E-04	6.67E-04	3.60E-04
					180	9.11E-06	0.00691	1.89E-07	1.07E-04	9.01E-04	4.05E-04
					200	5.28E-06	0.00684	9.18E-08	6.85E-05	1.03E-03	4.30E-04
	0.00045		0.01073	0.001	100	3.23E-05	0.01030	2.43E-06	4.10E-04	4.34E-04	4.18E-04
					120	2.96E-05	0.01021	1.47E-06	3.52E-04	6.22E-04	4.53E-04
					150	2.23E-05	0.01004	6.69E-07	2.52E-04	9.53E-04	5.15E-04
					180	1.30E-05	0.00987	2.70E-07	1.53E-04	1.29E-03	5.78E-04
					200	7.54E-06	0.00977	1.31E-07	9.78E-05	1.48E-03	6.15E-04
0.6	0.00018	10.468	0.00314	0.0003	100	1.82E-05	0.00299	1.26E-06	1.69E-04	1.37E-04	1.56E-04
					120	1.74E-05	0.00296	7.81E-07	1.50E-04	1.99E-04	1.68E-04
					150	1.44E-05	0.00290	3.82E-07	1.16E-04	3.13E-04	1.90E-04
					180	1.00E-05	0.00284	1.75E-07	8.02E-05	4.39E-04	2.15E-04
					200	6.82E-06	0.00279	9.61E-08	5.73E-05	5.21E-04	2.31E-04
	0.00030		0.00523	0.0005	100	3.03E-05	0.00499	2.10E-06	2.81E-04	2.28E-04	2.61E-04
					120	2.89E-05	0.00493	1.30E-06	2.50E-04	3.31E-04	2.80E-04

					150	2.41E-05	0.00484	6.36E-07	1.94E-04	5.21E-04	3.16E-04
					180	1.67E-05	0.00473	2.92E-07	1.34E-04	7.32E-04	3.58E-04
					200	1.14E-05	0.00466	1.60E-07	9.55E-05	8.68E-04	3.85E-04
					100	4.24E-05	0.00698	2.93E-06	3.94E-04	3.20E-04	3.65E-04
					120	4.05E-05	0.00691	1.82E-06	3.50E-04	4.64E-04	3.92E-04
	0.00042		0.00733	0.0007	150	3.37E-05	0.00677	8.90E-07	2.71E-04	7.29E-04	4.43E-04
					180	2.34E-05	0.00662	4.08E-07	1.87E-04	1.02E-03	5.01E-04
					200	1.59E-05	0.00652	2.24E-07	1.34E-04	1.21E-03	5.39E-04
					100	6.06E-05	0.00997	4.19E-06	5.63E-04	4.57E-04	5.21E-04
					120	5.79E-05	0.00987	2.60E-06	4.99E-04	6.63E-04	5.60E-04
	0.00060		0.01047	0.001	150	4.82E-05	0.00967	1.27E-06	3.87E-04	1.04E-03	6.32E-04
					180	3.34E-05	0.00945	5.83E-07	2.67E-04	1.46E-03	7.15E-04
					200	2.27E-05	0.00931	3.20E-07	1.91E-04	1.74E-03	7.70E-04

The names of the species associated to the alias presented in this table are collected in **Table 1**. Acronyms: $C_{i,\ initial}$: concentration of the species *i* at the inlet of the reactor. $C_{i,\ final}$: concentration of the species *i* at the outlet of the reactor.

Table S2

Concentration and flow rates of the species involved in the Aspen Plus 2nd Simulation (**Fig. 2**) at the inlet and outlet of the reactor.

$C_{ML, initial}$		$C_{ISOP, initial}$		Initial Flow Rate	T	$C_{ML, final}$	$C_{ISOP, final}$	$C_{INT, final}$	$C_{GVL, final}$	$C_{ACE, final}$	$C_{MOH, final}$	$C_{SUB1, final}$	$C_{SUB2, final}$	$C_{H2O, final}$
(mol/l)	(mol/min)	(mol/l)	(mol/min)	(l/min)	(°C)	(mol/min)	(mol/min)	(mol/min)	(mol/min)	(mol/min)	(mol/min)	(mol/min)	(mol/min)	(mol/min)
0.3	0.00009	10.987	0.00330	0.0003	100	7.59E-16	0.00312	8.25E-17	2.89E-08	9.08E-05	3.61E-12	6.01E-13	1.35E-04	9.00E-05
					120	1.47E-15	0.00312	1.16E-16	2.73E-08	1.19E-04	8.19E-12	5.21E-13	1.20E-04	9.00E-05
					150	2.91E-15	0.00312	1.62E-16	2.31E-08	1.66E-04	2.40E-11	3.74E-13	9.70E-05	9.00E-05
					180	4.46E-15	0.00312	1.85E-16	1.76E-08	2.13E-04	6.08E-11	2.34E-13	7.36E-05	9.00E-05
					200	5.36E-15	0.00312	1.81E-16	1.38E-08	2.42E-04	1.05E-10	1.57E-13	5.91E-05	9.00E-05
	0.00015		0.00549	0.0005	100	0.00E+00	0.00519	0.00E+00	4.81E-08	1.51E-04	6.02E-12	1.00E-12	2.24E-04	1.50E-04
					120	0.00E+00	0.00519	0.00E+00	4.55E-08	1.99E-04	1.37E-11	0.00E+00	2.00E-04	1.50E-04
					150	0.00E+00	0.00519	0.00E+00	3.85E-08	2.77E-04	4.01E-11	0.00E+00	1.62E-04	1.50E-04
					180	0.00E+00	0.00519	0.00E+00	2.94E-08	3.55E-04	1.01E-10	0.00E+00	1.23E-04	1.50E-04
					200	0.00E+00	0.00519	0.00E+00	2.31E-08	4.03E-04	1.75E-10	0.00E+00	9.86E-05	1.50E-04
	0.00021		0.00769	0.0007	100	0.00E+00	0.00727	0.00E+00	6.74E-08	2.12E-04	8.43E-12	1.40E-12	3.14E-04	2.10E-04
					120	0.00E+00	0.00727	0.00E+00	6.37E-08	2.78E-04	1.91E-11	1.22E-12	2.81E-04	2.10E-04
					150	0.00E+00	0.00727	0.00E+00	5.39E-08	3.87E-04	5.61E-11	0.00E+00	2.26E-04	2.10E-04
					180	0.00E+00	0.00727	0.00E+00	4.11E-08	4.96E-04	1.42E-10	0.00E+00	1.72E-04	2.10E-04
					200	0.00E+00	0.00727	0.00E+00	3.23E-08	5.64E-04	2.46E-10	0.00E+00	1.38E-04	2.10E-04
	0.00030		0.01099	0.001	100	0.00E+00	0.01039	0.00E+00	9.62E-08	3.03E-04	1.20E-11	2.00E-12	4.49E-04	3.00E-04
					120	0.00E+00	0.01039	0.00E+00	9.10E-08	3.98E-04	2.73E-11	1.74E-12	4.01E-04	3.00E-04
					150	0.00E+00	0.01039	0.00E+00	7.70E-08	5.53E-04	8.01E-11	1.25E-12	3.23E-04	3.00E-04
					180	0.00E+00	0.01039	0.00E+00	5.88E-08	7.09E-04	2.03E-10	0.00E+00	2.45E-04	3.00E-04
					200	0.00E+00	0.01039	0.00E+00	4.61E-08	8.06E-04	3.51E-10	0.00E+00	1.97E-04	3.00E-04
0.45	0.00013	10.728	0.00322	0.0003	100	0.00E+00	0.00295	0.00E+00	7.04E-08	1.14E-04	4.65E-12	1.86E-12	2.13E-04	1.35E-04
					120	0.00E+00	0.00295	0.00E+00	6.87E-08	1.51E-04	1.05E-11	1.67E-12	1.94E-04	1.35E-04

					150	0.00E+00	0.00295	0.00E+00	6.16E-08	2.14E-04	3.10E-11	1.30E-12	1.63E-04	1.35E-04
					180	0.00E+00	0.00295	0.00E+00	5.04E-08	2.81E-04	7.82E-11	0.00E+00	1.29E-04	1.35E-04
					200	0.00E+00	0.00295	0.00E+00	4.16E-08	3.25E-04	1.36E-10	0.00E+00	1.07E-04	1.35E-04
					100	0.00E+00	0.00491	0.00E+00	1.17E-07	1.89E-04	7.75E-12	3.09E-12	3.55E-04	2.25E-04
					120	0.00E+00	0.00491	0.00E+00	1.14E-07	2.52E-04	1.76E-11	2.79E-12	3.24E-04	2.25E-04
	0.00022		0.00536	0.0005	150	0.00E+00	0.00491	0.00E+00	1.03E-07	3.57E-04	5.16E-11	2.16E-12	2.71E-04	2.25E-04
					180	0.00E+00	0.00491	0.00E+00	8.40E-08	4.69E-04	1.30E-10	1.48E-12	2.15E-04	2.25E-04
					200	0.00E+00	0.00491	0.00E+00	6.93E-08	5.42E-04	2.26E-10	1.06E-12	1.79E-04	2.25E-04
					100	0.00E+00	0.00688	0.00E+00	1.64E-07	2.65E-04	1.09E-11	4.33E-12	4.97E-04	3.15E-04
					120	0.00E+00	0.00688	0.00E+00	1.60E-07	3.52E-04	2.46E-11	3.91E-12	4.54E-04	3.15E-04
	0.00031		0.00751	0.0007	150	0.00E+00	0.00688	0.00E+00	1.44E-07	5.00E-04	7.22E-11	3.03E-12	3.80E-04	3.15E-04
					180	0.00E+00	0.00688	0.00E+00	1.18E-07	6.57E-04	1.83E-10	2.07E-12	3.01E-04	3.15E-04
					200	0.00E+00	0.00688	0.00E+00	9.71E-08	7.59E-04	3.16E-10	1.49E-12	2.50E-04	3.15E-04
					100	0.00E+00	0.00983	0.00E+00	2.35E-07	3.79E-04	1.55E-11	6.18E-12	7.10E-04	4.50E-04
					120	0.00E+00	0.00983	0.00E+00	2.29E-07	5.03E-04	3.52E-11	5.58E-12	6.48E-04	4.50E-04
	0.00045		0.01073	0.001	150	0.00E+00	0.00983	0.00E+00	2.05E-07	7.15E-04	1.03E-10	4.32E-12	5.42E-04	4.50E-04
					180	0.00E+00	0.00983	0.00E+00	1.68E-07	9.38E-04	2.61E-10	2.95E-12	4.31E-04	4.50E-04
					200	0.00E+00	0.00983	0.00E+00	1.39E-07	1.08E-03	4.52E-10	2.12E-12	3.57E-04	4.50E-04
					100	0.00E+00	0.00278	0.00E+00	1.34E-07	1.33E-04	5.53E-12	4.18E-12	2.93E-04	1.80E-04
					120	0.00E+00	0.00278	0.00E+00	1.34E-07	1.77E-04	1.25E-11	3.87E-12	2.71E-04	1.80E-04
	0.00018		0.00314	0.0003	150	0.00E+00	0.00278	0.00E+00	1.24E-07	2.55E-04	3.68E-11	3.14E-12	2.32E-04	1.80E-04
					180	0.00E+00	0.00278	0.00E+00	1.06E-07	3.40E-04	9.30E-11	2.28E-12	1.90E-04	1.80E-04
0.6		10.468			200	0.00E+00	0.00278	0.00E+00	9.08E-08	3.98E-04	1.61E-10	1.71E-12	1.61E-04	1.80E-04
					100	0.00E+00	0.00463	0.00E+00	2.24E-07	2.21E-04	9.21E-12	6.97E-12	4.89E-04	3.00E-04
	0.00030		0.00523	0.0005	120	0.00E+00	0.00463	0.00E+00	2.23E-07	2.96E-04	2.09E-11	6.45E-12	4.52E-04	3.00E-04
					150	0.00E+00	0.00463	0.00E+00	2.07E-07	4.25E-04	6.13E-11	5.24E-12	3.87E-04	3.00E-04
					180	0.00E+00	0.00463	0.00E+00	1.77E-07	5.67E-04	1.55E-10	3.79E-12	3.16E-04	3.00E-04

					200	0.00E+00	0.00463	0.00E+00	1.51E-07	6.63E-04	2.69E-10	2.84E-12	2.68E-04	3.00E-04
					100	0.00E+00	0.00649	0.00E+00	3.14E-07	3.10E-04	1.29E-11	9.76E-12	6.85E-04	4.20E-04
					120	0.00E+00	0.00649	0.00E+00	3.12E-07	4.14E-04	2.92E-11	9.03E-12	6.33E-04	4.20E-04
	0.00042		0.00733	0.0007	150	0.00E+00	0.00649	0.00E+00	2.90E-07	5.95E-04	8.59E-11	7.34E-12	5.42E-04	4.20E-04
					180	0.00E+00	0.00649	0.00E+00	2.48E-07	7.94E-04	2.17E-10	5.31E-12	4.43E-04	4.20E-04
					200	0.00E+00	0.00649	0.00E+00	2.12E-07	9.29E-04	3.76E-10	3.98E-12	3.75E-04	4.20E-04
					100	0.00E+00	0.00927	0.00E+00	4.48E-07	4.42E-04	1.84E-11	1.39E-11	9.78E-04	6.00E-04
					120	0.00E+00	0.00927	0.00E+00	4.46E-07	5.92E-04	4.18E-11	1.29E-11	9.04E-04	6.00E-04
	0.00060		0.01047	0.001	150	0.00E+00	0.00927	0.00E+00	4.14E-07	8.51E-04	1.23E-10	1.05E-11	7.74E-04	6.00E-04
					180	0.00E+00	0.00927	0.00E+00	3.54E-07	1.13E-03	3.10E-10	7.58E-12	6.32E-04	6.00E-04
					200	0.00E+00	0.00927	0.00E+00	3.03E-07	1.33E-03	5.37E-10	5.69E-12	5.36E-04	6.00E-04

The names of the species associated to the alias presented in this table are collected in **Table 1**. Acronyms: $C_{i,\ initial}$: concentration of the species *i* at the inlet of the reactor. $C_{i,\ final}$: concentration of the species *i* at the outlet of the reactor.

Table S3
Concentration and flow rates of the species involved in the Aspen Plus 3rd Simulation (**Fig. 2**) at the inlet and outlet of the reactor.

$C_{ML, initial}$		$C_{ISOP, initial}$		Initial Flow Rate	T	$C_{ML, final}$	$C_{ISOP, final}$	$C_{INT, final}$	$C_{GVL, final}$	$C_{ACE, final}$	$C_{MOH, final}$	$C_{SUB1, final}$	$C_{SUB2, final}$
(mol/l)	(mol/min)	(mol/l)	(mol/min)	(l/min)	(°C)	(mol/min)	(mol/min)	(mol/min)	(mol/min)	(mol/min)	(mol/min)	(mol/min)	(mol/min)
0.3	0.00009	10.987	0.00330	0.0003	100	1.17E-06	0.00311	1.48E-07	2.59E-05	7.81E-05	7.20E-06	4.65E-10	9.95E-05
					120	8.31E-07	0.00310	7.29E-08	1.88E-05	1.07E-04	7.45E-06	3.22E-10	9.65E-05
					150	4.35E-07	0.00309	2.46E-08	1.09E-05	1.55E-04	7.73E-06	1.66E-10	8.51E-05
					180	1.96E-07	0.00308	7.95E-09	5.80E-06	2.05E-04	7.91E-06	7.42E-11	6.84E-05
					200	1.05E-07	0.00308	3.61E-09	3.62E-06	2.36E-04	7.98E-06	4.02E-11	5.64E-05
	0.00015		0.00549	0.0005	100	1.95E-06	0.00519	2.47E-07	4.32E-05	1.30E-04	1.20E-05	7.75E-10	1.66E-04
					120	1.38E-06	0.00517	1.22E-07	3.14E-05	1.78E-04	1.24E-05	5.37E-10	1.61E-04
					150	7.25E-07	0.00515	4.10E-08	1.82E-05	2.59E-04	1.29E-05	2.76E-10	1.42E-04
					180	3.26E-07	0.00513	1.33E-08	9.66E-06	3.42E-04	1.32E-05	1.24E-10	1.14E-04
					200	1.76E-07	0.00513	6.02E-09	6.03E-06	3.93E-04	1.33E-05	6.70E-11	9.40E-05
	0.00021		0.00769	0.0007	100	2.74E-06	0.00726	3.45E-07	6.05E-05	1.82E-04	1.68E-05	1.08E-09	2.32E-04
					120	1.94E-06	0.00724	1.70E-07	4.40E-05	2.49E-04	1.74E-05	7.52E-10	2.25E-04
					150	1.02E-06	0.00721	5.74E-08	2.54E-05	3.63E-04	1.80E-05	3.87E-10	1.98E-04
					180	4.57E-07	0.00719	1.86E-08	1.35E-05	4.78E-04	1.85E-05	1.73E-10	1.60E-04
					200	2.46E-07	0.00718	8.42E-09	8.44E-06	5.50E-04	1.86E-05	9.38E-11	1.32E-04
	0.00030		0.01099	0.001	100	3.91E-06	0.01038	4.94E-07	8.65E-05	2.60E-04	2.40E-05	1.55E-09	3.32E-04
					120	2.77E-06	0.01034	2.43E-07	6.28E-05	3.56E-04	2.48E-05	1.07E-09	3.22E-04
					150	1.45E-06	0.01030	8.20E-08	3.63E-05	5.18E-04	2.58E-05	5.52E-10	2.84E-04
					180	6.52E-07	0.01027	2.65E-08	1.93E-05	6.83E-04	2.64E-05	2.47E-10	2.28E-04
					200	3.51E-07	0.01026	1.20E-08	1.21E-05	7.86E-04	2.66E-05	1.34E-10	1.88E-04
0.45	0.00013	10.728	0.00322	0.0003	100	4.14E-06	0.00297	4.28E-07	5.27E-05	9.10E-05	1.02E-05	1.11E-09	1.37E-04
					120	3.25E-06	0.00295	2.28E-07	4.11E-05	1.27E-04	1.06E-05	8.45E-10	1.38E-04

					150	2.02E-06	0.00293	8.76E-08	2.67E-05	1.91E-04	1.12E-05	5.04E-10	1.30E-04
					180	1.08E-06	0.00291	3.24E-08	1.61E-05	2.62E-04	1.16E-05	2.65E-10	1.13E-04
					200	6.53E-07	0.00290	1.61E-08	1.09E-05	3.10E-04	1.18E-05	1.59E-10	9.75E-05
					100	6.91E-06	0.00495	7.14E-07	8.78E-05	1.52E-04	1.69E-05	1.85E-09	2.28E-04
					120	5.42E-06	0.00492	3.80E-07	6.85E-05	2.12E-04	1.77E-05	1.41E-09	2.30E-04
	0.00022		0.00536	0.0005	150	3.36E-06	0.00488	1.46E-07	4.45E-05	3.19E-04	1.86E-05	8.40E-10	2.17E-04
					180	1.80E-06	0.00485	5.40E-08	2.68E-05	4.37E-04	1.93E-05	4.41E-10	1.88E-04
					200	1.09E-06	0.00483	2.68E-08	1.82E-05	5.16E-04	1.96E-05	2.65E-10	1.62E-04
					100	9.67E-06	0.00693	1.00E-06	1.23E-04	2.12E-04	2.37E-05	2.60E-09	3.19E-04
					120	7.59E-06	0.00689	5.33E-07	9.58E-05	2.97E-04	2.47E-05	1.97E-09	3.22E-04
	0.00031		0.00751	0.0007	150	4.70E-06	0.00683	2.05E-07	6.23E-05	4.47E-04	2.60E-05	1.18E-09	3.04E-04
					180	2.52E-06	0.00678	7.56E-08	3.75E-05	6.12E-04	2.70E-05	6.17E-10	2.63E-04
					200	1.52E-06	0.00676	3.75E-08	2.54E-05	7.23E-04	2.74E-05	3.72E-10	2.27E-04
					100	1.38E-05	0.00990	1.43E-06	1.76E-04	3.03E-04	3.39E-05	3.71E-09	4.55E-04
					120	1.08E-05	0.00984	7.61E-07	1.37E-04	4.24E-04	3.53E-05	2.82E-09	4.60E-04
	0.00045		0.01073	0.001	150	6.72E-06	0.00975	2.92E-07	8.89E-05	6.38E-04	3.72E-05	1.68E-09	4.34E-04
					180	3.60E-06	0.00969	1.08E-07	5.36E-05	8.74E-04	3.86E-05	8.82E-10	3.75E-04
					200	2.18E-06	0.00966	5.35E-08	3.63E-05	1.03E-03	3.92E-05	5.31E-10	3.25E-04
					100	9.52E-06	0.00284	8.60E-07	8.33E-05	9.96E-05	1.28E-05	1.95E-09	1.65E-04
					120	7.98E-06	0.00281	4.82E-07	6.79E-05	1.41E-04	1.34E-05	1.57E-09	1.71E-04
	0.00018		0.00314	0.0003	150	5.55E-06	0.00278	2.02E-07	4.77E-05	2.17E-04	1.42E-05	1.03E-09	1.68E-04
					180	3.39E-06	0.00275	8.26E-08	3.15E-05	3.05E-04	1.49E-05	6.08E-10	1.53E-04
0.6		10.468			200	2.25E-06	0.00273	4.39E-08	2.28E-05	3.67E-04	1.53E-05	3.97E-10	1.38E-04
					100	1.59E-05	0.00473	1.43E-06	1.39E-04	1.66E-04	2.13E-05	3.24E-09	2.75E-04
	0.00030		0.00523	0.0005	120	1.33E-05	0.00469	8.03E-07	1.13E-04	2.35E-04	2.23E-05	2.61E-09	2.85E-04
					150	9.26E-06	0.00463	3.37E-07	7.96E-05	3.62E-04	2.37E-05	1.72E-09	2.81E-04
					180	5.66E-06	0.00458	1.38E-07	5.24E-05	5.08E-04	2.49E-05	1.01E-09	2.56E-04

					200	3.75E-06	0.00455	7.32E-08	3.79E-05	6.12E-04	2.55E-05	6.61E-10	2.30E-04
	0.00042		0.00733	0.0007	100	2.22E-05	0.00663	2.01E-06	1.94E-04	2.32E-04	2.98E-05	4.54E-09	3.85E-04
					120	1.86E-05	0.00656	1.12E-06	1.58E-04	3.29E-04	3.13E-05	3.65E-09	3.99E-04
					150	1.30E-05	0.00648	4.72E-07	1.11E-04	5.06E-04	3.32E-05	2.41E-09	3.93E-04
					180	7.92E-06	0.00641	1.93E-07	7.34E-05	7.12E-04	3.49E-05	1.42E-09	3.58E-04
					200	5.24E-06	0.00637	1.02E-07	5.31E-05	8.57E-04	3.57E-05	9.26E-10	3.21E-04
	0.00060		0.01047	0.001	100	3.17E-05	0.00947	2.87E-06	2.78E-04	3.32E-04	4.26E-05	6.49E-09	5.50E-04
					120	2.66E-05	0.00938	1.61E-06	2.26E-04	4.70E-04	4.47E-05	5.22E-09	5.70E-04
					150	1.85E-05	0.00925	6.74E-07	1.59E-04	7.23E-04	4.75E-05	3.44E-09	5.62E-04
					180	1.13E-05	0.00915	2.75E-07	1.05E-04	1.02E-03	4.98E-05	2.03E-09	5.11E-04
					200	7.49E-06	0.00910	1.46E-07	7.59E-05	1.22E-03	5.10E-05	1.32E-09	4.59E-04

The names of the species associated to the alias presented in this table are collected in **Table 1**. Acronyms: $C_{i,\ initial}$: concentration of the species *i* at the inlet of the reactor. $C_{i,\ final}$: concentration of the species *i* at the outlet of the reactor.

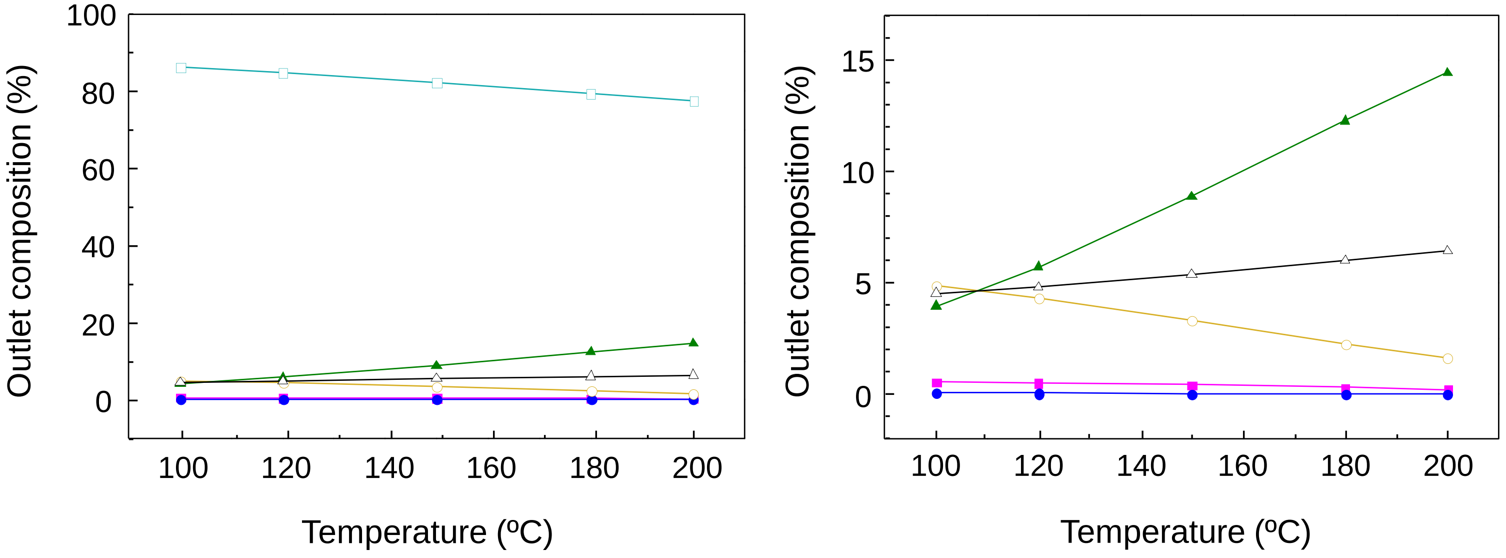

Fig. S1. Outlet stream composition (%) for the 1st simulation (**Fig. 2**) conducted in Aspen Plus at 35 bar by using a ML initial concentration of 0.6 mol·L^{-1} (regardless of the flow rate). Symbols: (-□-) ISOP, (-▲-) ACE, (-○-) GVL, (-△-) MetOH, (-■-) ML, (-●-) INT. The names of the species associated to these aliases are collected in **Table 1.**

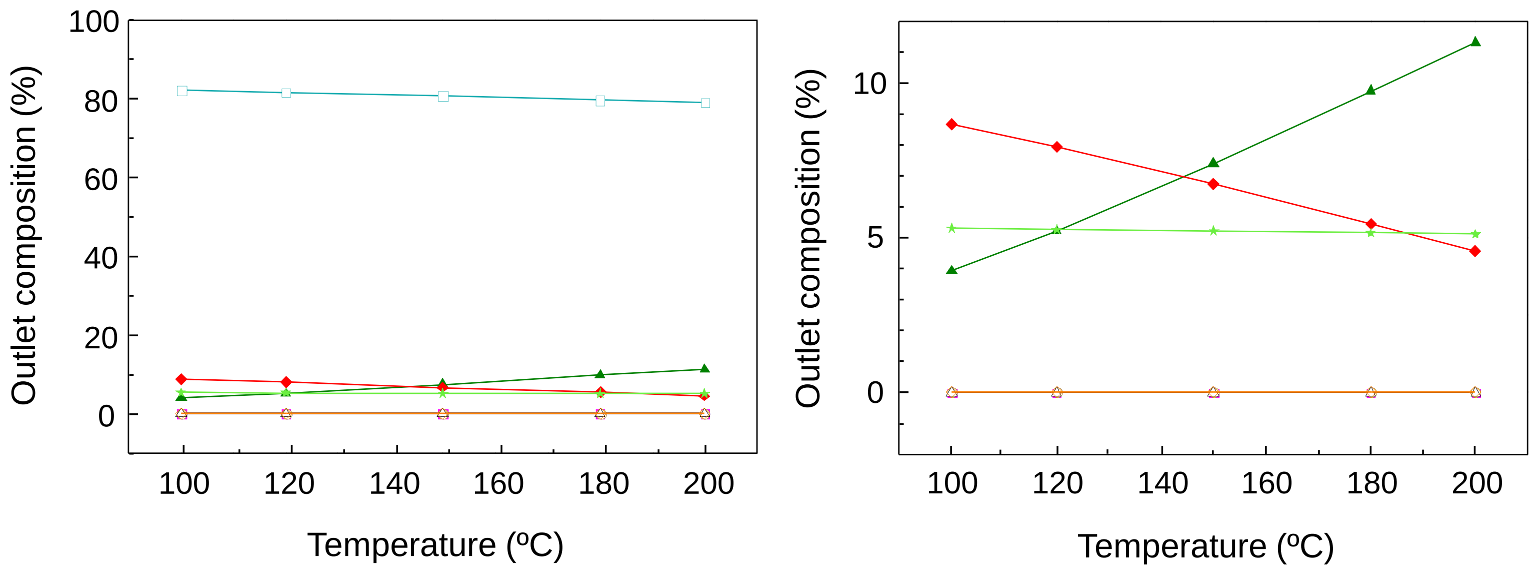

Fig. S2. Outlet stream composition (%) for the 2nd simulation (**Fig. 2**) conducted in Aspen Plus at 35 bar by using a ML initial concentration of 0.6 mol·L^{-1} (regardless of the flow rate). Symbols: (-□-) ISOP, (-▲-) ACE, (-◆-) BYP2, (-★-) H2O, (-○-) GVL, (-△-) MetOH, (-■-) ML, (-◇-) BYP1, (-●-) INT. The names of the species associated to these aliases are collected in **Table 1.**

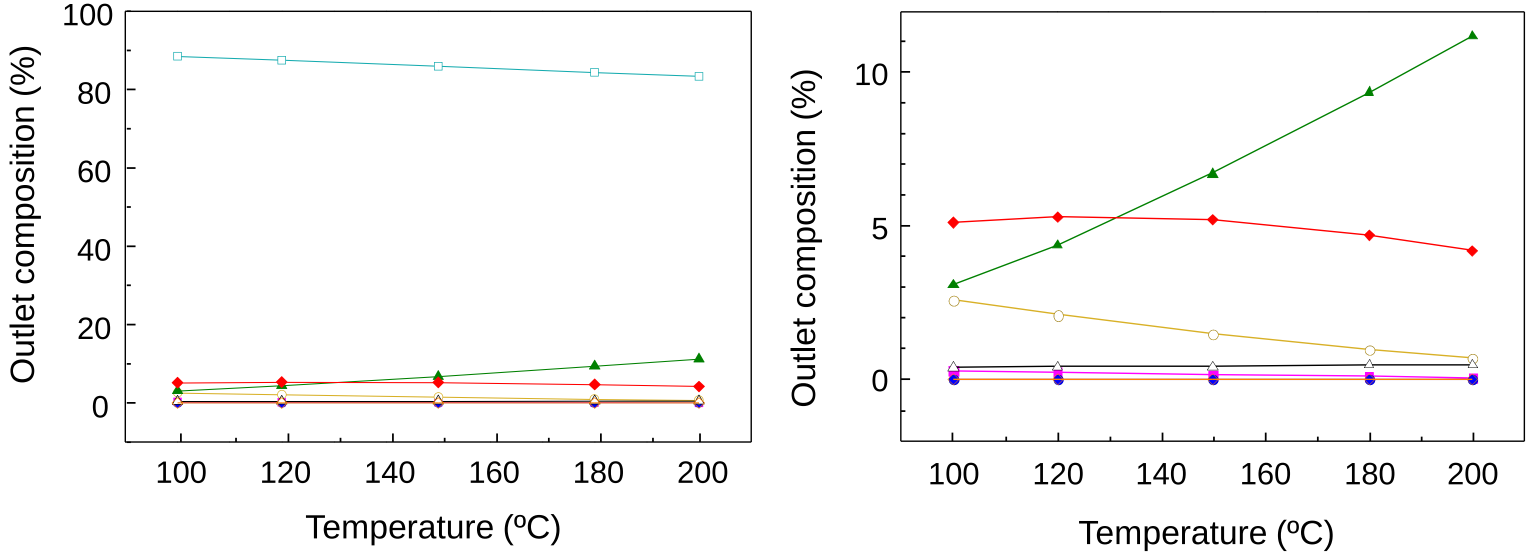

Fig. S3. Outlet stream composition (%) for the 3rd simulation (**Fig. 2**) conducted in Aspen Plus at 35 bar by using a ML initial concentration of 0.6 mol·L^{-1} (regardless of the flow rate). Symbols: (-□-) ISOP, (-▲-) ACE, (-◆-) BYP2, (-○-) GVL, (-△-) MetOH, (-■-) ML, (-◇-) BYP1, (-●-) INT. The names of the species associated to these aliases are collected in **Table 1.**
